# A Qualitative Analysis of the Use of Financial Services and Saving Behavior Among Older African Americans and Latinos in the Los Angeles Area

**DOI:** 10.1177/2158244014562388

**Published:** 2015

**Authors:** Luisa R. Blanco, Maria Ponce, Arturo Gongora, O. Kenrik Duru

**Affiliations:** 1 Pepperdine University, Malibu, CA, USA; 2 RAND, Santa Monica, CA, USA; 3 University of California, Los Angeles, USA

**Keywords:** social sciences, economic science, economic development, aging and the life course, sociology of health and illness, sociology of race and ethnicity, sociology

## Abstract

For this study, we conducted seven focus groups in the Los Angeles area with a total of 70 participants (42 Latinos and 28 African Americans) recruited from three senior centers and a church. There was a wide variety of responses in relation to the usage of financial services among participants. We found that although some participants seem to participate more in the formal financial sector and show a higher level of sophistication when managing their finances, other participants’ use of formal financial institutions is minimal. Among African American participants, we found several instances in which individuals feel very comfortable using banks. Lower levels of participation in the formal financial sector were found among the lower income Latino participants. In relation to barriers to participate in the financial sector, supply was not an issue, but demand and behavioral factors seem more important. Overall, no participants saved very much on a regular basis. We also find that participants in general do not want to ask their children for money, and also do not want to save and accumulate wealth to leave to their children.

## Introduction

There is a significant difference in participation in the formal financial sector, defined as having a checking account, by race and ethnicity among the elderly in the United States. When looking at data from the Health and Retirement Study (HRS; 2008 wave), it is observed that among adults 65 years and above, 41% of Latinos and 30% of African Americans do not participate in the formal financial sector. In contrast, only 7% of Whites 65 years and older do not participate in the financial sector. Participation in the formal financial sector here refers to ownership of a checking, savings, or money market account. Participation in the formal financial sector and the saving behavior of older minority groups is an important area of study because these groups face significant medical costs. Even those seniors with Medicare coverage need to pay deductibles and out of pocket health care expenses that represent a substantial amount of their income (Komisar, Kaiser Family Foundation Medicare Policy, & Henry J. Kaiser Family Foundation, 2012). It is also important to note that although some low-income individuals could have access to health services through Medicaid/Medi-Cal, dental care, vision services, speech therapy, and other services important for quality of life are not necessarily covered for these seniors (California Budget Project, 2013). As noted by Komisar et al. (2012), preparedness for future health expenses not covered by Medicare and Medicaid is related to socioeconomic status (SES), race, and ethnicity. Minority and low- and middle-income elders, particularly those not eligible for Medicaid, are less prepared.

We conducted a pilot study with the purpose of determining the use of financial services among older African Americans and Latinos, and how this participation is related to saving behavior, and their preparedness to cover health expenses not covered by Medicare/Medi-Cal. For this study, we conducted a qualitative analysis of focus group data from a convenience sample of 70 older Latinos and African Americans in the Los Angeles area. We hope to provide a deeper understanding on how older African Americans and Latinos participate in the formal financial sector and their saving behavior, which is not possible to do with a large data set such as the HRS.

We find that there was a wide variety of responses among the African American and Latino participants. Although some participants in the focus groups participate more in the formal financial sector and feel comfortable managing their finances, other participants’ involvement with the formal financial sector is minimal. Among the African American participants, there were several instances where we observed a high level of sophistication managing personal finances. Some participants only use a checking account to receive social security benefits and cash most of these benefits on a monthly basis. Lower levels of participation in the formal financial sector were found among some lower income Latino participants. Interestingly, we find that despite common perceptions, location, language barriers, and inability to talk with professionals in banks are not important barriers to participation in the financial sector. We find that most of the reasons given for not having a checking account are perceptions of high fees and distrust of financial institutions due to bad experiences with banks. Some individuals also expressed that they were not motivated to have a checking account because they were able to do all transactions in cash. In relation to saving, although some of the African American participants expressed that they are able to save on some occasions, no participants saved very much on a regular basis. Most participants expressed that they were unable to save on a regular basis because they never had enough income that would allow them to save on a regular basis.

We find that participants, regardless of income or race/ethnicity and Medicare and Medi-Cal coverage, are not well prepared for the future in terms of their ability to pay for uncovered health care expenses such as dental and long-term care, and out-of pocket health expenses such as premiums, deductibles, and cost sharing requirements. However, for some participants, particularly those with very low incomes, the decision to spend their limited resources on other cost-of-living expenses may make saving for future health care expenses impractical or impossible. Contrary to the general view that minorities will rely more on their children as they age, we find that most participants expressed that they do not want to ask their children for money and also do not want to save and accumulate wealth to leave to their children because they do not trust or feel appreciated by them. The article is organized as follows: we present first a background from the literature review, then we discuss the conceptual framework, and the description of the methodology, then we present the results, a discussion of results, and a conclusion.

## Participation in the Financial Sector, Saving Behavior, and Uncovered Health Care Needs Among the Elderly

It is important to understand the barriers to participation in the financial sector that older Latinos and African Americans face and the costs associated with not using financial services. A basic definition of participation in the formal financial sector is whether the individual has a checking account. There are factors from both the demand and supply sides that explain why ethnic and racial minorities are less likely to use financial institutions (Atkinson, 2006; Carbo, Gardener, & Molyneux, 2005; Commission of the European Communities, 2008). On the supply side, financial institutions might not be interested in offering services that address the needs of specific populations such as those with low income, the elderly, and immigrants (Jehoel-Gijsbers, Vrooman, & European Network of Policy Research Institutes, 2008). Therefore, financial institutions might not be interested in establishing bank branches in geographic areas where these populations live. Furthermore, financial institutions might not have the staff and resources available for communicating with non-English speakers. The financial instruments, specifically checking account services, offered by financial institutions might also not be adequate for these populations if these instruments are complex, have high fees, or require high balances.

On the demand side, SES and degree of assimilation into American society may explain the lack of participation in the formal financial sector. An individual’s occupation, income, and education are strongly correlated with the degree of participation in the formal financial sector (Claessens, 2006). Low financial literacy, which is related to low levels of education, also explains why some individuals use financial services to a lower degree (Cole & Nilesh, 2008). In relation to assimilation into American society and participation in the formal financial sector, research has mainly focused on Latino immigrants. Legal status, language, and long-term plans for residence affect the degree to which individuals use financial services, explaining why immigrants have lower usage of financial services (Barcellos, Smith, Yoong, & Carvalho, 2012; Datta, 2011). Cultural barriers, specifically those that relate to trust in banks, are also important for participation in the formal financial sector (Barcellos et al., 2012). Negative feelings about banks that could have resulted from bad experiences with these institutions can also affect the degree to which individuals use financial services.

Behavioral factors might also explain the lack of participation in the formal financial sector. According to Bertrand, Mullainathan, and Shafir (2004), it is important to consider not only those major factors related to rational models (cost of a bank account, financial education, etc.) but also small situational barriers (bus rides to a bank, challenging hours, interaction with bank personnel, etc.). Individuals will often weigh the costs and benefits of opening and using a checking account. As such, they might be unaware of the benefits of using the financial services offered or they might perceive that the cost of opening an account today is higher than the future benefits. In addition, an individual might lack the motivation to participate in the formal financial sector if he or she is part of the cash economy and wants to avoid declaring income for taxes or lacks a legal status to do so.

Participation in the formal financial sector is related to saving behavior, which consequently has an impact on the well-being and health outcomes of the individual. The connection between saving behavior and well-being derives from the fact that those individuals who are able and likely to save would be better prepared for future health expenses, and consequently, may be likely to have better long-term health outcomes. In relation to saving behavior, Lusardi and Michell (2007) developed the reasoning that non-planners have a lower probability of saving for the future. They found that individuals who slightly thought about saving were better off than their counterparts who did not think of saving at all. In addition, the accumulated retirement fund of an individual will be dependent on the income earned during their working years (Neelakantan & Chang, 2010). If individuals are not earning enough money, they will be less likely to focus on the later years and only have a short-term plan for their income. Automatic enrollment in saving plans has been believed to create a solution to the non-planner dilemma (Fisher & Anong, 2012). Prior studies (Poterba, Venti, & Wise, 1996; Samwick & Skinner, 2004) have shown that 401(k)s are generally better than defined benefit plans, but it can be difficult to enter due to eligibility limitations and higher investment fees. Access to 401(k)s plans also depends on the employer preferences and policies, which makes it not possible for some individuals to have these types of retirement plans even if they would like to have them.

Furthermore, financial literacy can have a large impact on the saving behavior of the elderly, where financial literacy is positively correlated with participation in the financial sector (Cole & Nilesh, 2008). Lusardi and Mitchell (2007) using the HRS data find that older African Americans and Latinos show lower levels of savings for retirement, which can be explained by lower financial literacy. In another study, Lusardi and Mitchell (2011) show that older Americans, women, minorities, and the least educated have low levels of financial literacy. The level of acculturation into the United States can also affect saving behavior, where immigrants tend to use financial services and save for the future less than the native-born population, where there are important language, institutional, and resource barriers (Fontes, 2011).

The lack of motivation to save, combined with low levels of financial literacy, has led to the use of short-term loans and familial relationships as buffers for emergency situations. Individuals are less likely to have a savings account for future emergencies and more likely to have money reserved in their checking accounts (Chase, Gjertson, & Collins, 2011). Preparing for retirement and any emergencies is also accompanied by the need to save for medical expenses in the present and future. Failing to plan for retirement, low-income levels, and increasing medical costs will greatly affect the ability of the elderly to afford uncovered health-related expenses in the future.

Although the great majority of adults 65 years and older are covered by Medicare, these beneficiaries still face significant health expenditures as they age, including out-of-pocket costs for covered health care as well as the full cost of uncovered health care. Many middle-class beneficiaries have insurance to supplement traditional Medicare, such as employer-sponsored health insurance, “Medigap” coverage, or membership in a Medicare Advantage plan with expanded benefits (Hoffman & Jackson, 2013). However, out-of-pocket expenses from deductibles, coinsurance, and prescription drug copayments can be substantial even for beneficiaries with supplemental coverage, and are increasing along with other health care costs. Although there is wide variation, median monthly health expenditures for a typical retiree without full Medicaid coverage were estimated at between US$215 and US$330 in 2010, and are expected to climb to between US$381 and US$571 in 2030 and between US$518 and US$788 in 2040 (Hoffman & Jackson, 2013). This pace of increase is projected to be more rapid than the increase in social security, the primary source of income for the majority of older adults.

Older adults also eligible for full Medicaid coverage (termed *dual-eligibles*) are sheltered from the majority of these costs, but such coverage is generally limited with some exceptions to the lowest-income Medicare beneficiaries (according to Families USA, 2013, with individual income less than approximately US$8,600 and individual assets less than US$2,000 in 2013). “Near-poor” beneficiaries with some supplemental coverage designed for low-income persons are required to contribute more to cover their health care expenses, and are at particularly high risk of adverse consequences. These beneficiaries tend to have little existing savings, and are likely to delay care, take out credit card debt or loans, and/or have difficulty paying for food, rent, or utility bills (Cubanski & Dulio, 2011; Komisar et al., 2012).

Minority retirees tend to have fewer resources and are more likely to face financial risks as they grow older. Estimates suggest that 34% of African American senior-headed households and 39% of Latino senior-headed households are considered to have “financial insecurity” strictly because of their health care expenses (Meschede, Wheary, Sullivan, & Shapiro, 2010). Among Latino and African American Medicare beneficiaries, the combination of lower median savings (US$9,800 and US$9,700 as compared with US$88,100 for Whites), a high incidence of current and future health complications, and increasing health care costs suggests impending problems for many (Komisar et al., 2012). Near-poor and middle-class minority retirees in particular face considerable economic turmoil from health and financial pressures that they may not fully understand, and would likely struggle to manage even if they are fully aware (Hoffman & Jackson, 2013).

These financial pressures are magnified further for older adults who live in areas with a high cost of living such as metropolitan Los Angeles. The cost-of-living index data from the 2010 Census include the cost of several essential goods and services—food, housing, utilities, transportation, and health care. On this index, the Southern California metro area, including Los Angeles, ranks as one of the eight most expensive areas to live in the United States, behind such locations as the New York metro area, the Bay Area of Northern California, the Washington, D.C. area, Hawaii, and Alaska (Census ref.). Older adults in these high-cost areas are likely to have major challenges coping with additional unexpected health-related expenses, which arise as they age.

## Conceptual Framework

When studying participation in the financial sector, saving behavior, and preparedness for future health expenses among older Latinos and African Americans, it is important to discuss the conceptual framework. The life-cycle model of savings is relevant to the current analysis. In this model, individuals are likely to smooth consumption through their entire lifetimes. It is expected that individuals will dissave early in life, then later, when they become active in the labor market, they will be able to save and accumulate wealth for retirement. Once the individual reaches retirement age, he or she will dissave again and draw on the funds saved while working (Modigliani, 1966).^1^ Under this model, it is expected that our participants, who are 65 years and older in the majority, have saved for retirement while they were working, and that currently, they are able to draw on the wealth accumulated over time. Under this model, there is also the possibility that individuals with very low incomes, particularly those who have saved little to date while raising families as middle-aged adults, may do precautionary saving in older age to cover high future health expenses or living expenses in later years. This model may be most relevant to individuals who participate in the formal financial sector, because the accumulation of wealth for retirement would be more secure in this sector as compared with the informal financial sector.

Second, based on the review of the literature, participation in the financial sector is also dependent on supply, demand, and behavioral factors. In relation to supply factors, availability of financial institutions, adequate financial services, and personnel with language skills and training to address the needs of different populations are relevant when determining participation in the financial sector. From the demand side, SES and cultural barriers are relevant. Behavioral factors related to the cost–benefit analysis under-taken by the individual when deciding whether to have a checking or saving account are also important determinants of participation in the financial sector.

## Method

We conducted seven focus groups with older Latinos and African Americans in the Los Angeles area. Each focus group had between 8 and 12 participants, with a total of 70 participants. Sixty percent of the participants were Latinos and 40% were African Americans in our sample (42 Latinos and 28 African Americans).^2^ Individuals were recruited using convenience sampling from three senior centers (Florence-Firestone, Willowbrook, and Culver City Senior Centers) and one church (Guardian Angel Church of Pacoima). We conducted three focus groups with older African Americans and four focus groups with older Latinos. The focus groups lasted approximately 90 min, and a short survey was administered to gather demographic characteristics of the participants and information about the use of financial services, saving behavior, access to care, and well-being.

We decided to focus our study on older Latinos and African Americans in the Los Angeles area for a few reasons. First, analysis of data from the HRS, which is nationally representative, showed that participation in the financial sector among older Latinos and African Americans is significantly lower in comparison with Whites of the same age group. These differences in participation are still significant across different racial and ethnic groups, even after controlling for individual characteristics related to SES such as income, wealth, and education (Blanco, Aguila, Leng, & Angrisani, 2014). Second, using a convenience sample of older Latinos and African Americans in different senior centers in the Greater Los Angeles area provided us with a sample of individuals who live in a major city, which is relevant when trying to understand participation in the financial sector among urban populations in the cities.

Although we do not have specific individual information about income for all the participants, we believe that it is possible to generalize and classify participants in three main groups: (a) African Americans (AA; Culver City and Willowbrook participants), (b) middle-income Latinos (MIL; Culver City participants), and (c) low-income Latinos (LIL; Florence-Firestone and Pacoima participants). According to the information provided by the directors of the senior centers, center members in Willowbrook have an annual average income between US$14,400 and US$24,000, which can be considered middle-income. However, members in the Florence-Firestone center have an annual average income between US$7,000 and US$11,000, which is below poverty level and can be considered low-income. The director from the Culver City Senior Center informed us that they do not collect any type of information in relation to income among members, but Culver City is a middle/upper class community in West Los Angeles. As for the participants from Pacoima, individuals were recruited from members of the Guardian Angel Catholic Church. This church is located in the middle of a large, public low-income housing complex, which allows us to infer that these participants can be considered in the low-income group.

The focus groups with African American participants were conducted in English and those with Latino participants were conducted in Spanish. All instruments for this study were developed in English and Spanish, and the facilitator who conducted all the focus groups was bilingual. All focus groups were recorded and transcribed, and the transcriptions in Spanish were translated into English by two bilingual researchers.

The main topics for discussion in the focus groups centered on the research questions of this study:

**Research Question 1:** What is the degree of participation of older Latinos and African Americans in the formal financial sector?

**Research Question 2:** What is the saving behavior among older Latinos and African Americans and how is this related to their ability to cover health-related expenses?

We applied the *Scissor-and-Sort Technique* and *Content Analysis* to analyze the focus group data.^3^ We first developed a codebook that allowed us to categorize all the information in the transcriptions relevant for this study.^4^ To check for consistency in the coding, two researchers coded a portion of the same transcription and the two principal investigators (PI) reviewed the coded data. After going through the same transcription, researchers and PIs met to discuss any inconsistency in the data coding and any modifications needed for the codebook. The coding of this transcription was very similar, where researchers coded in the same way around 90% of the time. The two researchers coded the rest of the transcriptions separately (one researcher coded half of the transcriptions, whereas the other coded the other half, approximately), and this coding was supervised by the PIs. After the data were coded, the researchers cut and pasted together the relevant information into categories. Three documents that contained the coding for the following groups were created: (a) AA, (b) MIL, and (c) LIL. The PIs went through these documents and performed a content analysis, which is discussed in the next section. Input from the researchers was also obtained for the content analysis section.

In relation to basic sample characteristics, 19% were 60 to 64 years old, 21% were 65 to 69, 41% were 70 to 79, 11% were 80 to 89, and 3% were 90 and above.^5^ Fifty-seven percent of the participants were born outside the United States, where 46% speak Spanish at home, 11% speak English and Spanish equally at home, and 43% speak English at home.

## Results

### Survey Results

There are some interesting differences across groups that are observed from the data collected through the short survey conducted among the focus groups participants.^6^ Table 1 presents information from the survey for the full sample and for the three subgroups of interest discussed above (AA, MIL, and LIL) related to participation in the formal financial sector, saving behavior, access to care, and well-being.^7^ In relation to the SES of the full sample, Latino participants show lower levels of education than African Americans. We also observed that the LIL participants show lower degree of home ownership than the other groups.

In relation to participation in the formal financial sector, among those who answered the question, 76% of the participants had a checking account, whereas 24% did not. Fifty-three percent had a savings account, 47% did not. Furthermore, only 25% of the participants are able to save on a monthly basis. In relation to credit, 43% owned a credit card, whereas 57% did not. Table 1 shows that the degree of participation and saving behavior varies across groups. Lack of participation in the financial sector was more prominent among LIL, where less than half of this population has a checking account (48%). For the AA and MIL participants, ownership of a checking account is 100% and 89%, respectively. Whereas only 27% of the LIL participants have a savings account, 56% and 83% of the MIL and AA have a savings account, respectively. Saving on a monthly basis was more common among AA participants, where we observe that 64% of this group is able to save on a monthly basis, whereas among MIL and LIL participants, we observe that only 11% and 3% can save on a regular basis, respectively.

Furthermore, we observe for the full sample that more than two thirds of the participants receive social security benefits (69%), but only around one fourth of the participants have a pension account (24%). When comparing groups, there is not a stark difference in relation to access to social security benefits among participants, but ownership of a pension for retirement is more common among AA participants than among LIL participants (55% versus 3%). When talking about access to care, most of the participants have health insurance (89%). Nonetheless, 70% and 60% have no coverage for expenses related to orthodontics and ophthalmology, respectively. Lack of coverage for these expenses seems to be higher for LIL participants than AA participants.^8^

### Focus Group Results—Participation in the Formal Financial Sector

Our data show that there is a wide range of experiences in terms of participation in the formal financial sector among minority elders. Although some participants seem to show higher levels of sophistication when dealing with their finances and using financial services, other participants use banks to a lower degree, and others do not use banks at all. The term of sophistication refers here to the degree to which participants understand how banks and financial instruments work, how they make use of financial services, and how they carry their personal finances. High levels of sophistication refer to those individuals who have a better understanding of financial services, make use of different financial instruments, and carefully keep track of their personal finances. We observed from the qualitative data that there were many AA participants with high levels of sophistication when dealing with their finances. We find that whereas most participants from the MIL group use financial services on a regular basis, most participants from the LIL group use financial services to a minimal level or do not have participation in the financial sector at all. We discuss further the degree to which responses vary in our study in relation to participation in the financial sector and some of the factors explaining this variation.

The following direct quotations from our transcriptions of the focus groups show the wide range of responses we received in relation to participation in the formal financial sector:

I like my bank. I never had no problems. I have been with them 35 years. I never had no problem with them. (AA Participant)

I barely opened an account because of the letter that beginning March they would not send the [social security] check home, that I had to open a bank account. (LIL Participant)

When I was younger I worked taking care of children, but I never opened an account. Everything that they paid me was in cash. (LIL Participant)

In relation to physical access to financial services, geography and availability of financial institutions do not seem to be a barrier in our study. Participants overall reported that there is a large number of formal financial institutions in their neighborhood, and that they generally have no trouble getting to them in most cases. In very few instances, it was mentioned that they needed to take the bus or ask a family member for a ride to get to their bank. We observed that most participants were able to mention about three to six banks or credit unions in their neighborhood. Latino (middle- and low-income) participants generally showed a high degree of awareness of alternative financial services, such as local check cashing places, pawnshops, and short-term loans places, and of the availability of these services. Most Latinos in our sample mentioned using these alternative services at least once, whereas only few African Americans in our sample mentioned using them and only in a few instances. We also found that there is always staff that speaks Spanish in most financial institutions, and that most participants feel comfortable interacting with the staff that works in those places. Few LIL participants expressed that in some instances, they were willing to use alternative financial services specifically because they had transportation issues that precluded them from going the bank.

In relation to participation in the formal financial sector, we observed a wide range of responses. We noted a high level of participation in the formal financial sector among most AA participants. Most of them mentioned in the focus group discussion that they have had an account in the bank for a long time because they opened it when they started working early in their life. Although specific questions about financial literacy were not part of the discussion in the focus groups, AA participants seem financially literate. Some of the participants in this group mentioned that they used several financial instruments, and many of them expressed that they keep track of their account balances religiously. From the conversations, we observe that many participants from this group had a good understanding of how banks work.

In our study, African American participants seem comfortable participating in the formal financial sector. However, although they show some degree of trust in financial institutions, many are skeptical and “keep an eye on them.” Although participants in this group have different attitudes toward banks, they are aware of the fees that the banks charge them, and if they had a problem with their bank, they moved to another bank. Some people in this group use online banking to check their balances, which was not the case with the other groups. In relation to alternative financial services, such as check cashing places and pawnshops, most AA participants in this sample strongly dislike them and have used them on very few occasions. Many AA participants perceive that the high fees these places charge are a “rip-off.” In general, most AA participants seem to be very engaged and active in their personal money management with the banks. As we saw in the results from the survey, all AA participants reported having a checking account (100%), and the conversations during the focus groups corroborated this.^9^

However, we observed lower levels of participation in the financial sector among the Latino participants, for the most part. Although most of MIL and some of LIL participants have bank accounts, their degree of interaction with the formal financial sector is minimal. Most MIL participants have checking accounts, write checks, and use their debit card regularly. From the discussion, we also observed that there is a wide distribution among MIL participants in terms of having a savings account, and very few LIL participants have a savings account. Thus, although AA and MIL participants would look relatively the same in terms of participation in the financial sector if we focused only on whether they have a checking account, MIL participants are less likely to have savings accounts and therefore use financial services to a lower degree.

Many participants from the LIL group show very low levels of participation and usage of financial services. Most LIL participants mentioned that they use their checking accounts for the sole purpose of receiving social security benefits, not to write checks. Most participants use direct deposit for social security benefits, but many LIL cash their social security checks every month and they do not write checks or use a debit card against these accounts. Several LIL participants mentioned that they opened a checking account only when they started receiving social security benefits, and that they never had a bank account before. We observed another subset of LIL participants who do not have a bank account and furthermore do not see the need for one. Those who do not have a bank account in our sample are likely to be those who do not get social security benefits. Being part of the cash economy, where one is paid only in cash and all economic transactions are also done with cash, is one reason why some LIL participants do not want to open a checking account.

As with African Americans, Latinos in our sample, regardless of income, seem to be very concerned about the fees that the banks charge and minimum balance requirements. To some degree, it seems as if LIL participants in particular face high fees when accessing the formal financial sector, or at least have a perception that fees are high specifically from having a checking account. It could be the case that this group keeps lower balances, which leads banks to charge them high fees. It could also be the case that this group is unaware of the different financial services available that might allow them to reduce the fees they will pay for these services, such as keeping a minimum balance, opening a saving account, or using direct deposit. Some LIL participants report that they banked in Latin America but do not bank in the United States because of the fees and confusing fine print associated with bank accounts in the United States.^10^ Some LIL participants also mentioned that they closed their accounts if they had a problem with the bank, instead of switching to another bank as some African Americans did. These concerns about bank fees were reported by MIL participants but less frequently. Thus, it could be inferred that the difference in responses among MIL and LIL in general in relation to the perception of bank fees is likely to be related to class.

In an apparent contradiction, despite concern about high fees charged by banks, many LIL participants in particular are using alternative financial services and paying the fees associated with those services. In several instances, LIL participants mentioned that sometimes, they have transportation issues because they do not own a car, and this motivates them to use the alternative financial services that are available in their locality. Some LIL participants use the check cashing places and Western Union. They also mentioned that they like to use cash or money orders to pay their bills. They feel that using cash is more convenient, and some expressed that they feel more secure using money orders to pay their bills. Among LIL, there seems to be a greater acceptance of the fees that go along with alternative financial services as compared with fees charged by banks, and one participant notes that these fees “are a part of life.” Some LIL participants found it more convenient to use the alternative financial services than to use banks, despite the fees associated with these services. Nonetheless, there were a minority of LIL participants who think that the fees associated with alternative financial services are too high, and that is why they prefer to use banks.

In relation to trust in banks, we observed that distrust of banks is high among Latino participants regardless of income. In the MIL group, several participants mentioned that they do not feel their money is safe in banks, but that banks are a better alternative than the “mattress.” There seem to be several instances among LIL participants where they report that the bank deliberately “took money from them” in what they perceived as a form of fraud. Several LIL participants provided stories about how the bank kept the money they had in their account or how they suffered identity theft, lost the money they had in the bank, and were not able to recover it. These perceptions of bank “fraud” were not raised in the MIL or AA groups.

In summary, we found several factors that explain why participation in the formal financial sector among many older Latinos in our study is low. For those participants without a checking account or who use financial services to a minimal degree, the main factors influencing their low level of participation in the financial sector seemed to be their lack of understanding of financial services (i.e., low levels of financial literacy), preference for using cash, and distrust in banks. Low levels of financial literacy are likely to be correlated with educational levels. Furthermore, there were several individuals among the LIL participants who stated that they did not know how to read and write, which might also explain their inability to understand financial services. Finally, 93% of the Latinos in the sample were born outside the United States, and although most of them have been in the United States for a long time (before the 1990s), the culture of banking does not seem to be ingrained among the participants from this group.

Other reasons to not participate in the financial sector also expressed among those who do not have a checking account, and that are related to behavioral factors, are the perception of a lack of both money and motivation to open an account. Many LIL participants expressed that they do not have the money to keep a balance in the bank, and therefore, they perceive they would be charged high fees for keeping the account open. Some LIL participants also mentioned that they simply did not have enough motivation to go to open an account or that they have not opened one due to procrastination.

### Focus Group Discussion Results—Saving Behavior and Health

Although some participants in each of the focus groups own a savings account, this was more common among the AA group. Interestingly, several AA and MIL participants expressed that they are able to save on a regular basis. Whereas the AA group participants typically mentioned that they will save in a savings account, many individuals in the MIL group said that they will save in their checking account or in cash. However, most participants from the LIL group expressed that they do not have a savings account and that they are unable to save on a regular basis due to their low income. Most LIL participants say that they have never been able to save.

Several AA participants seemed to be able to save on a monthly basis, and the need to save for a “rainy day” was brought up many times in the discussion, which was not the case in the discussion with the other groups. For example, an AA participant describes the idea of having a rainy day fund:

You live from day to day. But once … you know, you always hear your poor parents say—Put a little money away for a rainy day. And I used to hear them say that, but I didn’t have money for a rainy day. But as the time went on, I realized that what they were saying is always put money back in case of an emergency or in case you needed it or in case you just weren’t working and you need it, so you had something to lean on. So that’s why I started saving early on.

Furthermore, in the AA group, there were several cases where people saved through different financial instruments (CDs and mutual funds), and many participants expressed that they started saving very young. Some participants in this group also mentioned that they like to transfer money from their checking to savings account on a monthly basis. However, when we discussed employer-based saving plans for retirement, such as the 401(k) or 401(b), we found that most participants had never had retirement accounts, and were therefore not able to draw from those accounts during retirement or for a “rainy day.” Among the Latino participants, we also observed that saving for retirement or for a rainy day is very low or nonexistent. Most Latinos in our study had never been able to save, and also did not have retirement accounts to draw from. One LIL participant expressed the following:

I do not save. There is no saving. We are broke. So save, no, because my employment did not allow it and I did not earn much. What I worked—I would not have enough to sustain my family.

We also found that in general, regardless of race or income levels, most participants are not saving in order to offset future health expenses that are not covered through Medicare and Medi-Cal. This is despite the fact that many of them have chronic conditions, are at risk of significant illness, and are likely to accrue significant uncovered health-related costs in terms of long-term care and other uncovered health expenses. Although saving behavior does vary by group, no one seems to be saving for future health expenses. The majority of the participants have Medicare and/or Medi-Cal and are very aware that their current health costs are mainly covered. Due to the strict asset test required of Medi-Cal beneficiaries who are 65 years and above, these individuals have little incentive to save.

We discussed with participants two potential scenarios: (a) Participants would need to pay US$800 for dentures, and (b) participants will need to pay US$400 for a serious illness that leads to health expenses not covered by Medicare and Medi-Cal. These situations differed in that the first can be considered discretionary spending, although high-quality dentures are important for adequate food intake as well as social interactions (Walls, Mysore). The second can be considered essential spending.

In the case of essential spending for serious illness, which could lead to potentially uncovered expenses, most of them answered that they would go to a county hospital to get whatever services they need and either pay in installments, find assistance with reducing the bill, or just not pay at all. In the scenario given, we specify to them that they will need to pay US$400 for the health expenses not covered by Medicare and Medi-Cal. Although some participants mentioned that they would establish a payment plan to pay for these expenses, others responded that they would not pay because they do not have the money and would choose to “be a burden on the system.” Some of the Latino participants are also aware of a program in Los Angeles County called Outpatient Reduced–Cost Simplified Application (ORSA) that covers patients who do not have legal status, at least for outpatient care. Here is an example of how participants said they will pay for uncovered care:

“It depends on what area you live in. Because there are county hospital[s] and you become a burden to the state … . me, I would be a burden.” “I would have to ask for payment, set up a payment plan or something. Because I couldn’t afford $400 right there and then.” (AA Participants)

Well they give you a deduction. Two years ago I did not have social security. I had to go because I had a really bad pain in my back and they had to take me to the general hospital. They had me for 10 days. They never sent me a receipt, a year later they send it. It was 36,000 dollars … but I went and they reduced it to 1,000 dollars. Still a lot of money for me. But I paid them in hundreds. In payments. (MIL Participant)

“If I did not have an insurance or the money, I would go to a big hospital, Luther King, General, and then after they took me in and billed me I would go to Orchard [ORSA] and ask for their help. Since I do not have the economic resources, that is what I would do.” “I would say that I do not have a way to pay and I would tell them, look I cannot pay but I can pay little by little. That is my concept, if I did not have a plan.” “[Moderator] But none of you have a friend or relative or you children that could pay the 400 dollars for the emergency? There is no one that will lend. There is no one that will help you.” (LIL Participants)

In relation to the discretionary scenario where individuals have to pay for dentures, we found a similar situation. For this scenario, we told participants that they needed to pay US$800 for the dentures, and most participants said that they would not have enough savings to cover this and would likely not be able to afford them. Most participants said they would try to work with the dentist to set up a payment plan so that they can make small monthly payments. Surprisingly, no participant mentioned that they would ask their children for this money.

Although few participants are saving for future uncovered health expenses, several participants noted that they are currently paying or saving for their eventual funeral, so that family members do not have to cover these expenses. Saving or paying for funeral expenses was something that came up especially among the LIL participants.

From the overall discussion, it is evident that participants are not extensively worried about their future health expenses not covered by Medicare or Medi-Cal. One of the reasons is that most participants had access to care either through Medicare or Medi-Cal, and their copayments for doctor visits, medicines, and preventive care are very small. To the extent that participants are aware of free or low-cost support services for vulnerable populations with health care needs, they may decide to put their limited financial resources toward other cost-of-living expenses instead, such as housing and transportation. Another reason is that these individuals might be unaware of what future health expenses are not covered by Medicare or Medi-Cal, and therefore, do not feel the need to save for these expenses. This behavior may actually be appropriate for patients with Medi-Cal who may lose their coverage if they exceed the savings limit established by the assets test. However, no participants raised this issue of losing insurance coverage as a disincentive to their own savings behavior, and the lack of savings behavior may not be an intentional response to these eligibility issues.^11^

### Focus Group Discussion Results— Intergenerational Relationships and Financial Behavior

Another interesting and somewhat unexpected finding from the focus groups relates to the nature of the financial and care-giving relationship between older Latinos and African Americans with their children. Many older adults save to cover expenses they will encounter as they continue to age, and also to be able to leave money to their children and grandchildren. Children and grandchildren are often expected to provide care for the older adult when they may have difficulty with self-care. The general consensus is that minorities may particularly depend on their children more than Whites to provide informal care as they age, as they may have fewer assets to pay for formal care (Cantor, Brennan, & Sainz, 1994; Mendes de Leon & Glass, 2004). Our study provided data that conflict with this view of intergenerational relationships, as we noted that many of the participants in this study do not seem to trust their children very much with money, or in general. For example, several participants from each of our groups expressed frustrations such as the following:

“You leave them houses and whatever … And then they don’t appreciate it … And one year after you’re gone, boom … . It’s all gone … . They can’t wait till you die if they know you got some [money].” “No. I’m spending that inheritance now. And anything left after I’m gone, then this is what they’ll have. But I’m not going to save my money for somebody if something happened to me that they’re not going to even appreciate what I did.” (AA Participants)

So I do not need to go ask them for this or that. On my part, I am responsible of taking care of two properties. If I keep asking them [for help] they tell me: “What do you do with all your money? Why can’t you do it?” (MIL Participant)

I was always a mother who liked to save money for her children. Now, I have 20 grandchildren, I got disappointed so badly that I said, “No more.” I have to live for today. Of course I watch my pennies, but no more saving for no one because no one appreciates the sacrifices that are or were made. So instead I enjoy it. (LIL Participant)

Based on our focus groups, it seems that if participants really need financial assistance and/or informal care from their children in the future, it may create friction. There are variations in the relationships with children in all groups, with perhaps better parent–child relationships in the MIL group overall compared with the others. Nonetheless, several participants in all groups expressed that they do not want to save and accumulate wealth to leave to their children because they do not trust or feel appreciated by them. This is also an interesting finding about intergenerational wealth transmission among older minorities. Nonetheless, it is important to note that some participants did mention to have a good relationship with their children. Thus, the degree to which participants rely on family networks is not uniform.

## Discussion

Our study contributes to the literature in several important ways. Our focus group discussions allow us to better understand what the barriers are to participation in the formal financial sector and how they relate to the supply, demand, and behavioral factors discussed in the literature review. It was interesting to find out that behavioral factors are important determinants of the lack of participation in the financial sector among many participants from the Latino group. Although it was thought initially that supply side factors might be relevant explaining the lack of participation, we found that availability of banks and bilingual staff was not an issue in most cases. Nonetheless, one issue that needs to be studied further is whether the financial instruments available for this population are adequate.

We find that the demand and behavioral factors are more relevant explaining the lack of participation in the formal financial sector among many Latino participants. Among those participants who do not have a checking account, their perception about the required minimum balance and bank fees is what keeps them from opening an account. The issue of assimilation into American society is also relevant in explaining low usage of financial services among Latino participants. We observed that several LIL participants did not see the need to open a checking account because they have been part of the cash economy. The issue of trust is something that also seems to explain low levels of participation. Although most groups express some distrust in financial institutions, we observed that LIL participants seemed more averse to banks and provided many examples in which they had bad experiences with banks. These bad experiences with banks are likely to be associated with their higher levels of distrust. Most LIL participants feel more comfortable using cash and alternative financial services for their everyday transactions, and this behavior was not observed as often among participants from the other groups.

There seems to be a connection between financial sophistication and saving behavior, where more sophisticated participants are more likely to save, but we found that most participants are not well prepared to face unexpected expenses associated with uncovered care. When looking at the issue of saving among these groups, one could argue that there are valid reasons that justify why they are not saving. Because most participants are in the middle- and low-income range, and are dependent on social security benefits, it is not possible for them to save. Some participants expressed that the income they receive from social security benefits, which is their main and unique source of income, is barely enough to cover their monthly expenses. Others also mentioned that they never had enough income that would allow them to save on a regular basis. Thus, their inability to save is tied to their low levels of income. Our study was conducted in Los Angeles, a very expensive metropolitan area in which many people are unable to even afford standard cost-of-living expenses such as housing and transportation. However, we had expected that some middle-income participants in Los Angeles might be able to save for future health expenses, but we found that this was generally not the case.

Another argument why it is rational for these individuals not to save is related to the life-cycle hypothesis. Most of the participants are now retired or are unable to be economically active. Under the life-cycle hypothesis, it is expected that these individuals have saved while they were young so that they have accumulated wealth that could be used when they retire or become economically inactive. Nonetheless, as the data from the survey show, very few individuals in the sample have saving and retirement accounts. Finally, some of our study population had Medi-Cal insurance. Medi-Cal beneficiaries are not incentivized to save as they could lose their insurance coverage if they exceed an assets threshold.

The reasons provided above can be used as arguments justifying why it is rational for minority elders not to save. However, relying on a fixed income and not having any funds to cover health expenses that are not covered by Medicare and Medi-Cal are likely to result in financial insecurity among these populations. This may be inevitable given the lack of pre-retirement savings in this population and the high cost of living, but is not ideal. For those patients not at risk of loss of Medi-Cal due to assets testing, finding a way to save for future expenses would be advantageous. According to a guide from National Council on Aging (2011), there are ways in which older adults can save. Participation in the formal financial sector is likely to allow seniors to better prepare for their future expenses. Actions such as having a savings account that draws interest, managing personal finances to avoid fees, and avoiding alternative financial services that charge high fees, can help minority elders to save. Thus, promoting the participation and usage of financial services among minority elders through educational programs might be beneficial. In fact, some participants expressed their interest on participating in an educational program on personal finances.

Our research provides some insights into the potential uptake and effectiveness of a new program proposal. In January 2014, President Obama announced his intent to roll out the My Retirement Account (myRA) savings plan that targets low-income and middle-income households who would otherwise rely on social security once retirement occurred. The White House (2014b) explains that the myRA savings plan would focus on increasing savings by creating an easy to use and easy to access retirement account. Functioning similarly to a Roth Individual Retirement Account (IRA), myRA plans would encourage employees to participate with small, automatic, and voluntary contributions deducted from each paycheck with low risks and increased security. The plan would mainly target employees who earn less than US$191,000 per year and where employers do not already administer or contribute to a retirement account (The White House, 2014a). The contributions would only be invested in government savings bonds. As a result, the rate of return would be relatively low as compared with private funds, but the government would also be able to guarantee that none of the initial principal would be lost.

Employers would have the option to offer myRA plans to employees without any administrative costs and contribution commitments (The White House, 2014a). Administrative costs would be avoided due to enrollment into the program being offered online for the employee to complete, and because the program would be managed by the government. Individuals would not be charged any fees to participate. Initial contributions could be as low as US$25, and subsequent automatic payroll deductions could be as low as US$5 per pay period (The White House, 2014b). Individuals would not deposit large portions of their income into accounts for future use, increasing the likelihood of additional saving in the present.

Although our study focused on adults 65 years and above, some of our findings are useful when projecting how this program may be received by the target population of low- and middle-income working age adults. The absence of fees will be very important in creating acceptance among this population, as our work shows that transaction or “low balance” fees charged by financial institutions are a frequently cited and important issue for middle- and low-income minority adults. Some of our participants understood the details of the fees but had strong objections, whereas others interpreted the fees as deliberate fraud by the financial institution, but both groups tended to switch or leave the financial institution charging the fees. In addition, our participants would likely be disinclined to participate in a privately held retirement fund that could drop in value based on the stock market. The myRA program, to the extent that it will clearly communicate the absence of fees and the guarantee of retaining all principal invested, may be able to increase saving for retirement among low-to-middle income African American and Latino adults.

Another interesting finding from this study that requires further discussion is the relationship between participants and their children. As discussed before, most participants expressed that they do not want to rely on their children to cover unexpected health expenses that are not covered by Medicare and Medi-Cal. This is an important finding as it shows us that minority elders, as they age and are affected by serious illness, are a particularly vulnerable population. Our finding here goes against the traditional view that Latinos and African Americans are more likely to rely more on their children as they age (Cantor et al., 1994).^12^ To our knowledge, there is little current research showing that there is a change in the trend on intergenerational social support among older Latinos and African Americans.

It is important to note some of the limitations to this study. First, this study was conducted with limited resources among a small convenience sample, which limits the ability to generalize to all participants in the broader population as well as to substratify the focus groups by variables such as insurance status. Second, our sample is geographically limited to the Los Angeles area, and may be less relevant to older adults in smaller cities, suburbs, or rural areas, particularly those that have a lower cost of living. Third, our analysis is entirely qualitative because we use data obtained from focus groups. Our pilot study provides us with some insights into how individuals behave, which might be difficult to obtain from larger surveys, but we cannot claim that our findings have statistical significance. Fourth, we did not include White older adults or low-income African American older adults in our sample. Thus, we cannot comment on racial/ethnic disparities between minorities and Whites, or consistently relate distinctions between themes specifically to class versus race/ethnicity. It is expected that middle-income Whites might behave similarly to middle-income African Americans. It will be interesting to study whether the behaviors observed among elderly low-income Latinos are similar to low-income Whites or low-income African Americans. Finally, we were not able to collect individual financial information for the participants. Many of the participants were not willing to provide such personal details, and the senior centers did not want us to risk alienating their clients by insisting on this.

## Conclusion

Participation in the formal financial sector has become an important area of research, but there is little attention to the degree to which older minorities participate in the formal financial sector and the implications of this participation in their saving behavior. Our qualitative analysis allow us to better understand human behavior because we are able to discuss directly with individuals the reasons why or why not they use financial services, whether they are able to save on a regular basis, and whether they are prepared for unexpected future health expenses not covered by Medicare and Medi-Cal. To our knowledge, there are no secondary data that provide us with this information. Our qualitative analysis can provide the foundation for the development of a future survey that can be used to study participation in the financial sector and saving for retirement behavior among minorities in the United States.

An important finding relevant for policymaking from this study is that minority elders are likely to be unprepared to face unexpected health expenses that are not covered by Medicare and Medi-Cal. This is not entirely unexpected and may be a rational response given other economic pressures, but is less than ideal for these populations as they continue to age. From the data collected, we also found that minority elders are not able to rely on family members to cover unexpected health expenses, which is new to the literature. This provides a challenge for the health care system as this group ages. Thus, understanding the magnitude of this problem is recommended for future research. We also recommend further research to understand the behavioral factors that make minority elders less likely to participate in the formal financial sector, especially among older Latinos because this is the group in which we observed lower levels of participation in our study.

## Figures and Tables

**Table 1:** Survey Results (%^a^).

	African American	Latinos	Latinos	Full
	(*n* = 28)	Middle income (*n* = 10)	Low income (*n* = 32)	Sample (*n* = 70)
Education				
Elementary not completed	4	63	53	33
Elementary completed	0	0	20	9
Middle school completed	0	0	13	6
High school completed	64	38	3	33
College completed	32	0	10	18
Living status				
Owned, with mortgage	44	63	22	35
Owned, without mortgage	24	13	9	15
Rent for cash	32	25	66	48
Occupied without payment of rent	0	0	3	2
Checking account				
Have one	100	89	52	76
Do not have one	0	11	48	24
Do not know	0	0	6	3
Do not want to answer	14	10	0	7
Savings account				
Have one	83	56	27	53
Do not have one	17	44	73	47
Do not know	0	0	7	3
Do not want to answer	28	10	11	18
Saving behavior				
Able to save in a monthly basis	64	11	3	25
Not able to save in a monthly basis	36	89	97	75
Do not know	0	0	0	0
Do not want to answer	21	10	0	10
Credit card				
Have one	58	89	17	43
Do not have one	42	11	83	57
Do not know	0	0	6	3
Do not want to answer	14	10	0	7
Social security				
Receive social security benefits (self, partner, both)	78	63	65	69
Do not receive social security benefits	22	38	35	31
Do not know	0	0	0	0
Do not want to answer	15	20	0	9
Pension for retirement^b^				
Have a pension (self)	55		3	24
Do not have a pension	45		97	76
Do not know	0		3	2
Do not want to answer	20		3	11
Health insurance				
Have health insurance	96	100	80	89
Do not have health insurance	4	0	20	11
Do not know	0	0	6	3
Do not want to answer	8	10	0	4
Orthodontics^b^				
Have coverage	45		20	30
Do not have coverage	55		80	70
Do not know	0		0	0
Do not want to answer	13		0	6
Ophthalmology^b^				
Have coverage	57		29	40
Do not have coverage	43		71	60
Do not know	0		0	0
Do not want to answer	13		0	5
Health self-assessment				
Excellent	8	29	6	9
Very good	20	14	6	13
Good	32	14	13	20
Fair	28	43	69	50
Poor	12	0	6	8
Do not know	0	0	0	0
Do not want to answer	4	30	0	6
Life satisfaction				
Completely satisfied	33	50	16	26
Very satisfied	29	50	32	33
Somewhat satisfied	33	0	32	30
Not very satisfied	0	0	13	7
Not at all satisfied	4	0	6	5
Do not know	0	0	3	1
Do not want to answer	8	40	0	9

a Percentages for the answers *Do not know*/*Do not want to answer* were calculated out of the total number of answers. Percentages for the other answers are calculated out of the total of those that have answered the question (number of people who answer *Do not know*/*Do not want to answer* is not considered when these percentages are calculated).

b Question was added to survey later in the study, not available for some groups.

## Footnotes

1 See Modigliani (1966) for a discussion on the life-cycle hypothesis.

2 Two African Americans, who participated in the African American focus groups, identified themselves also as Hispanics. Because these two individuals seem to identify themselves stronger with the African American group in the recruitment process, we include them in the African American group for the sample description.

3 For further explanation of these techniques, please refer to Krueger and Casey (2008) and Neuendorf (2002).

4 Codebook and other instruments of the study are available on request.

5 Two people left this question blank and one person disclosed at the end of the focus group that she was in her 50s (4%).

6 In our discussion of the results, we do not include direct quotations from the focus groups for purpose of space in most cases. A working version of this article, which is available online, includes direct quotations from focus groups.

7 Percentages for the answers *Do not know/Do not want to answer* were calculated out of the total number of answers. Percentages for the other answers are calculated out of the total of those who have answered the question (number of people who answer *Do not know/Do not want to answer* is not considered when these percentages are calculated).

8 These questions were added to the survey later in the study and that is why we do not have observations for all groups. In fact, the question was added to the survey as a result of the first focus group with middle-income Latinos (MIL), where the discussion of this uncovered care came up.

9 The 100% is calculated among those who answered the question. Among African Americans (AA), 14% did not want to answer the question.

10 From the focus group discussion, it is unclear whether the individuals faced different fees in Latin American banks.

11 California Department of Health Care Services provides a table showing income levels required for qualifying for Medi-Cal (http://www.dhcs.ca.gov/services/medi-cal/Pages/DoYouQua-lifyForMedi-Cal.aspx).

12 Refer to Mendes de Leon and Glass (2004) for a good literature review on social support among minority elders.
